# *In vitro *study on the schedule-dependency of the interaction between pemetrexed, gemcitabine and irradiation in non-small cell lung cancer and head and neck cancer cells

**DOI:** 10.1186/1471-2407-10-441

**Published:** 2010-08-19

**Authors:** An Wouters, Bea Pauwels, Filip Lardon, Greet GO Pattyn, Hilde AJ Lambrechts, Marc Baay, Paul Meijnders, Jan B Vermorken

**Affiliations:** 1 Laboratory of Cancer Research and Clinical Oncology, Department of Medical Oncology, University of Antwerp, Universiteitsplein 1, 2610 Wilrijk, Belgium; 2 Department of Radiotherapy, University Radiotherapy Antwerp (URA), Lindendreef 1, 2020 Antwerp, Belgium

## Abstract

**Background:**

Based on their different mechanisms of action, non-overlapping side effects and radiosensitising potential, combining the antimetabolites pemetrexed (multitargeted antifolate, MTA) and gemcitabine (2',2'-difluorodeoxycytidine, dFdC) with irradiation (RT) seems promising. This *in vitro *study, for the first time, presents the triple combination of MTA, dFdC and irradiation using various treatment schedules.

**Methods:**

The cytotoxicity, radiosensitising potential and cell cycle effect of MTA were investigated in A549 (NSCLC) and CAL-27 (SCCHN) cells. Using simultaneous or sequential exposure schedules, the cytotoxicity and radiosensitising effect of 24 h MTA combined with 1 h or 24 h dFdC were analysed.

**Results:**

Including a time interval between MTA exposure and irradiation seemed favourable to MTA immediately preceding or following radiotherapy. MTA induced a significant S phase accumulation that persisted for more than 8 h after drug removal. Among different MTA/dFdC combinations tested, the highest synergistic interaction was produced by 24 h MTA followed by 1 h dFdC. Combined with irradiation, this schedule showed a clear radiosensitising effect.

**Conclusions:**

Results from our *in vitro *model suggest that the sequence 24 h MTA → 1 h dFdC → RT is the most rational design and would, after confirmation in an *in vivo *setting, possibly provide the greatest benefit in the clinic.

## Background

During the past decades, the use of chemotherapeutic drugs in combination with radiotherapy has become a common strategy for the treatment of different solid tumours, improving cancer care dramatically. Combining chemotherapy and radiotherapy has a solid and appealing biologic rationale. Firstly, whereas radiotherapy is aimed at controlling the primary tumour, the cytotoxic drug also eradicates distant micrometastases, a scenario defined as spatial cooperation. Secondly, chemotherapy can possibly enhance the effects of ionising radiation, a process called radiosensitisation [1].

The availability of several new active compounds has led to the development of promising new combinations. Many of those chemoradiotherapy combinations include antimetabolites, because of their efficacy, their generally well-defined mechanisms of action and mostly manageable toxicities [2]. In this *in vitro *study, we describe for the first time the triple combination of the antimetabolites gemcitabine and pemetrexed with irradiation in two human tumour cell lines.

Gemcitabine (2',2'-difluorodeoxycytidine, dFdC) is a synthetic pyrimidine nucleoside analogue clinically active against a wide variety of solid tumours. Transport of gemcitabine across the plasma membrane is mostly mediated by the human equilibrative nucleoside transporter 1 (hENT1) [3]. Intracellularly, the prodrug gemcitabine requires phosphorylation and hence activation by deoxycytidine kinase (dCK). The diphosphate (dFdCDP) and triphosphate (dFdCTP) forms of the drug are presumed to be responsible for the cytotoxic effect, as they inhibit ribonucleotide reductase or are incorporated into the DNA, leading to chain termination, respectively [4].

In addition to its cytotoxic effect, gemcitabine has potent radiosensitising properties, as shown in both preclinical and clinical settings [5]. The current evidence suggests that accumulation in the S phase of the cell cycle, depletion of dATP pools, reduction of apoptotic threshold, inhibition of DNA synthesis and reduction of DNA repair might contribute to, or might even be essential for gemcitabine-mediated radiosensitisation [6-10]. Recently, Pauwels et al could grant a role for cell cycle perturbations and activation of the extrinsic apoptotic pathway in the radiosensitising effect of gemcitabine [10]. On the other hand, it has been suggested that radiosensitisation by gemcitabine may be primarily explained by the significant inhibition of DNA repair following combined radiation and gemcitabine treatment. DNA repair pathways using short DNA patches, such as non-homologous end joining and base excision repair, are thought not to play an important role in gemcitabine-mediated radiosensitisation [9,11]. Instead, homologous recombination, a long-patch DNA repair pathway, has been argued to be the target for gemcitabine to enhance cellular radiosensitivity [9]. Moreover, the role of the mismatch repair (MMR) system, an intermediate-patch DNA repair pathway, may be of relevance [12]. A dramatic increase of nucleotide misincorporations in gemcitabine-treated (MMR deficient) cells was demonstrated, presumably due to dNTP pool imbalances (particularly dATP depletion) [8,13]. Van Bree et al showed that MMR proficiency reduced radiosensitisation after 24 h incubation with a low dose of gemcitabine, suggesting that the MMR status might affect the recovery from gemcitabine treatment [14].

Pemetrexed (multitargeted antifolate, MTA) is a new-generation antimetabolite with antitumour activity against a broad range of human malignancies [15]. It was approved by the FDA for first-line treatment of inoperable malignant mesothelioma in combination with cisplatin [16]. Successively, pemetrexed was also investigated in non-small cell lung cancer (NSCLC), where it was FDA-approved as second-line therapy in patients with previously chemotherapy-treated advanced NSCLC [17], as first-line therapy, in combination with cisplatin, for chemotherapy-naive NSCLC patients [18], and, very recently, for maintenance treatment of patients with locally advanced or metastatic non-squamous NSCLC whose disease had not progressed after four cycles of platinum-based first-line chemotherapy [19].

Pemetrexed acts as a multitargeted antifolate by inhibiting multiple key enzymes involved in both pyrimidine and purine synthesis, its primary targets being thymidylate synthase (TS), dihydrofolate reductase (DHFR) and glycinamide ribonucleotide formaldehyde transferase (GARFT). Pemetrexed enters the cell mainly by a reduced folate carrier system. Once inside the cell, pemetrexed is an excellent substrate for the enzyme folylpolyglutamate synthase (FPGS) [20], which rapidly converts pemetrexed to its active polyglutamate derivatives that have a substantially higher potency for inhibition of TS and GARFT [21]. It is believed that polyglutamation of pemetrexed plays a profound role in determining both the selectivity and the antitumour activity of this agent.

The ability of pemetrexed to deplete cellular nucleotide pools, to modulate the cell cycle, and to induce apoptosis makes this drug an attractive cytotoxic agent for polychemotherapy regimens and combination with radiotherapy [22]. In preclinical studies, radiosensitisation by pemetrexed was observed in human colon, breast, cervix and lung carcinoma cells [23]. *In vivo*, combination of pemetrexed with fractionated radiotherapy produced additive to greater than additive antitumour activity in murine and human tumour xenografts [24,25]. In a phase I study, it was suggested that pemetrexed could be administered at systemically active doses in combination with radiotherapy [26]. These findings prompted further investigation of the radiosensitising effect of pemetrexed.

The aim of the present study is the exploration of the cytotoxic (and not toxic) effects of combinations of pemetrexed and gemcitabine alone or combined with irradiation using various treatment schedules in two human carcinoma cell lines. Given the three approved indications for pemetrexed in the treatment of NSCLC, we selected the A549 NSCLC cell line. As radiotherapy in combination with gemcitabine is reported to be feasible and highly active in the treatment of locally advanced squamous cell carcinoma of the head and neck (SCCHN) [27], we also included the CAL-27 SCCHN cell line.

## Methods

### Cell lines

The cell lines used in this study were CAL-27 (squamous cell carcinoma of the tongue) and A549 (lung adenocarcinoma). A549 was cultured in RPMI-1640 medium, supplemented with 10% dialysed foetal calf serum, 2 mM glutamine and 1 mM sodium pyruvate. CAL-27 was cultured in DMEM medium, supplemented with 10% dialysed foetal calf serum and 2 mM glutamine (Invitrogen, Merelbeke, Belgium). No antibiotics were used. Cells were grown as monolayers and cultures were maintained in exponential growth in a humidified 5% CO_2_/95% air atmosphere at 37°C. For subsequent experiments, cells were collected by trypsinisation, counted, and plated as specified below. The cell doubling times of A549 and CAL-27 cells in our experimental conditions were 28 h and 33 h, respectively.

### Cytotoxicity experiments

Cells were plated in 48 well plates and seeding densities were 500 cells/cm^2 ^for A549 and 1400 cells/cm^2 ^for CAL-27, assuring exponential growth. Cells were incubated with pemetrexed alone (0-2000 nM for 24 h), gemcitabine alone (0-100 nM for 24 h or 0-5 μM for 1 h) or with a combination of both, where one drug was used at a fixed concentration, while a concentration range of the other drug was added. Three combination schedules were tested: (1) simultaneous exposure to pemetrexed and gemcitabine for 24 h; (2) gemcitabine for 1 h or 24 h immediately followed by pemetrexed for 24 h; (3) pemetrexed for 24 h immediately followed by gemcitabine for 1 h or 24 h. Cell survival was determined by the sulforhodamine B (SRB) assay, as previously described [28]. The experimental conditions adopted in this study, including time of exposure to gemcitabine and pemetrexed, are similar to those selected in previous studies [10,22,29,30]. In simultaneous experiments, treatment exposure time was 24 h. To reduce the variation in drug efficacy associated with time of drug addition relative to plating time, the sequential schemes examined the effect of both a 1-h and a 24-h gemcitabine exposure on the cytotoxicity of pemetrexed. Since several clinical studies have indicated that administration of gemcitabine and pemetrexed immediately after each other is well tolerated and clinically active [31-33], no wash out with drug-free medium was included in the sequential treatment schedules.

### Chemoradiation experiments

Cells were plated in 48 well plates, with plating densities assuring exponential growth during the experiments. The radiosensitising effect of pemetrexed alone was investigated by including different time intervals (24 h, 8 h, 4 h, 1 h, 0 h) between 24 h pemetrexed treatment and irradiation (0-8 Gy, room temperature, linear accelerator (URA, Antwerp)). The same time intervals as in a previous study investigating the schedule-dependency of gemcitabine treatment combined with radiation were included [34]. In additional experiments, cells were first irradiated, immediately followed by 24 h incubation with pemetrexed. The triple interaction between pemetrexed, gemcitabine and irradiation was investigated including the three combination schedules previously mentioned, immediately followed by irradiation.

After 7 or 8 days, survival was determined by the SRB assay. For *in vitro *radiosensitivity testing, this method was comparable to the clonogenic assay, taking into account some critical aspects [28].

### Cell cycle experiments

Cells were plated in 6-well plates and incubated with 50 nM (CAL-27) or 100 nM (A549) pemetrexed for 24 h. Cell cycle analysis was performed 0, 1, 4, 8 or 24 h after drug wash out (referred to as 24+0, 24+1, 24+4, 24+8 and 24+24 respectively). Hence, cells were treated with pemetrexed using the same treatment schedules as adopted in the pemetrexed-radiotherapy experiments, but without subsequent radiation. As such, cell cycle perturbations at the time of irradiation were measured. Cell cycle distribution was monitored according to the Vindelov method, as described previously [34]. Samples were analysed using a FACScan flow cytometer (Becton Dickinson). Histograms of DNA content were analysed using FlowJo software to determine the fractions in each phase of the cell cycle (G_0_/G_1_, S and G_2_/M). The Watson-Pragmatic model was used to fit curves to the stages of the cell cycle [35].

### Statistical analysis

All experiments were performed at least three times. The results are presented as mean ± SD. Possible significant differences (p < 0.05) were evaluated with two-sample t-tests and two-way ANOVA, using SPSS v16.0 software.

Survival rates were calculated by: [mean optical density (OD) of treated cells/mean OD of control cells] × 100%. Radiation dose-survival curves were fitted according to the linear-quadratic model using WinNonlin software (Pharsight, Mountain View, USA) with survival = exp(-αD-βD^2^). The radiation dose-survival curves were corrected for the cytotoxic effect of the drugs alone. The following parameters were calculated: IC_50 _(drug concentration causing 50% growth inhibition); ID_50 _(radiation dose causing 50% growth inhibition); SF_2 _(surviving fraction at 2 Gy); and MID (mean inactivation dose, calculated by numeric integration of the linear-quadratic curve [36]). The radiosensitising effect was represented by the dose enhancement factor (DEF), calculated as the ID_50 _for control, untreated cells divided by the ID_50 _for the treated cells.

The interaction between pemetrexed, gemcitabine and/or radiation was analysed using CalcuSyn software (Biosoft, Cambridge, UK) to determine possible synergism. Data from the cell survival curves were expressed as the fraction of cells killed by the individual drugs or the combination in drug-treated cells compared with untreated cells. The CalcuSyn program is based upon the Chou-Talalay method, which calculates the combination index (CI). The analysis is performed based on the following equation: CI = (D)_1_/(D_x_)_1 _+ (D)_2_/(D_x_)_2 _where (D)_1 _and (D)_2 _are the doses (or concentrations) of drug 1 and drug 2 that have x% effect when used in combination and (D_x_)_1 _and (D_x_)_2 _are the doses of drug 1 and drug 2 that have the same x% effect (i.e. isoeffect) when used alone. (D_x_)_1 _and (D_x_)_2 _can be readily calculated from the median-effect equation of Chou: D_x _= D_m _[f_a _/(1-f_a_)]^1/m^, where f_a _is the fraction affected, D_m _is the median-effect dose (IC_50 _or ID_50_) that inhibits the system under study by 50% and m is the coefficient signifying the sigmoidicity of the dose-effect relationship [37]. The CI values obtained from the classic (mutually exclusive) isobologram calculations were used. CI < 1.0, CI = 1.0 and CI > 1.0 indicate synergism, additivity or antagonism, respectively. Moderate synergism is depicted by CI values between 0.7 and 0.9, synergism by CI values below 0.7.

## Results

### Cytotoxicity of pemetrexed alone

A clear concentration-dependent cytotoxic effect of pemetrexed was observed in CAL-27 and A549 cells, with IC_50 _values of 118.77 ± 17.28 nM and 629.89 ± 68.77 nM respectively. The cytotoxicity of pemetrexed was greatly dependent on the cell line used, as for example in the PANC-1 pancreatic cell line, no dose-response relationship was observed and concentrations up to 1500 μM induced only 30% cell kill.

### Combining pemetrexed with radiation

To investigate potential interactions between pemetrexed and radiation therapy, CAL-27 and A549 tumour cells were exposed to various doses of pemetrexed for 24 h (0-100 nM for CAL-27; 0-500 nM for A549), immediately followed by irradiation (0-8 Gy) (table 1). No concentration-dependent radiosensitising effect of MTA was observed in CAL-27 cells, with DEF values around 1.00 for all concentrations tested. CI analysis showed that 24 h incubation with MTA immediately before irradiation resulted in moderate antagonism (1.1<CI<1.3). In the A549 cell line, DEFs slightly decreased with an increasing concentration of MTA. The mean CI varied from 0.689 ± 0.079 to 0.887 ± 0.294, indicating synergism to additivity.

**Table 1:** DEF, CI, ID_50_, SF_2 _and % survival (representing the cytotoxic effect of treatment with MTA alone, 0 Gy) for irradiation (RT) given alone or in combination with MTA using different treatment schedules: 24 h MTA immediately followed by RT; RT immediately followed by 24 h MTA; 24 h MTA followed by different time intervals (24, 8, 4, 1, 0 h) and subsequent irradiation.

	Condition	DEF			CI			**ID**_**50**_			**SF**_**2**_			% survival	
		mean	SD		mean	SD		mean	SD		mean	SD		mean	SD
**CAL-27**	RT							1.94	0.47		48.37	9.31		100%	0%
	24 h 50 nM MTA → RT	1.04	0.23		1.145	0.132		1.97	0.45		49.25	9.71		97%	11%
	24 h 75 nM MTA → RT	1.01	0.10		1.260	0.270		1.94	0.54		47.75	7.53		70%	12%
	24 h 100 nM MTA → RT	1.04	0.03		1.229	0.269		1.86	0.27		47.34	4.31		57%	4%
															
	RT → 24 h 50 nM MTA	0.87	0.06		1.039	0.098		1.86	0.46		46.21	9.19		88%	4%
	RT → 24 h 75 nM MTA	0.94	0.14		1.023	0.063		1.77	0.63		44.56	12.48		71%	8%
	RT → 24 h 100 nM MTA	1.00	0.24		1.005	0.080		1.65	0.41		42.40	10.00		59%	12%
															
	24 h 50 nM MTA → 24 h → RT	1.59	0.53	†	0.915	0.063		1.71	0.66		43.37	12.35		70%	21%
	24 h 50 nM MTA → 8 h → RT	1.23	0.31		0.844	0.091	*	1.55	0.58		39.45	12.88		88%	4%
	24 h 50 nM MTA → 4 h → RT	1.12	0.24		0.965	0.106		1.68	0.58		42.34	12.02		90%	6%
	24 h 50 nM MTA → 1 h → RT	1.46	0.21	†	0.932	0.073		1.21	0.35	†	32.45	8.43	†	98%	4%

**A549**	RT							2.41	0.39		56.26	6.18		100%	0%
	24 h 100 nM MTA → RT	1.29	0.25		0.698	0.079	**	1.77	0.36		45.39	7.15		87%	11%
	24 h 200 nM MTA → RT	1.19	0.06		0.855	0.197	*	2.18	0.34		52.16	4.46		65%	12%
	24 h 300 nM MTA → RT	0.97	0.23		0.887	0.294	*	2.84	1.03		57.46	8.70		52%	14%
	24 h 500 nM MTA → RT	0.75	0.28		0.793	0.288	*	3.83	1.56		62.76	10.23		38%	13%
															
	RT → 24 h 200 nM MTA	0.86	0.19		0.996	0.124		2.77	0.20		60.24	3.01		68%	19%
	RT → 24 h 300 nM MTA	0.80	0.10		0.862	0.173	*	2.98	0.30		61.48	2.65		52%	16%
	RT → 24 h 500 nM MTA	0.76	0.11		0.723	0.166	*	3.11	0.19		62.75	1.09		38%	15%
															
	24 h 100 nM MTA → 24 h → RT	0.98	0.15		0.832	0.067	*	2.16	0.11		52.70	2.00		84%	16%
	24 h 100 nM MTA → 8 h → RT	1.21	0.18		0.731	0.129	*	2.02	0.24		50.62	4.87		85%	9%
	24 h 100 nM MTA → 4 h → RT	1.56	0.41		0.718	0.103	*	1.63	0.35		42.19	6.30		94%	10%
	24 h 100 nM MTA → 1 h → RT	1.52	0.39		0.677	0.076	**	1.85	0.51		46.28	9.78		85%	11%

†: p < 0.05 vs. 24 h incubation with the same concentration of MTA immediately followed by RT; *: moderate synergism (0.7<CI<0.9); **: synergism (CI<0.7).

**Figure 1:**
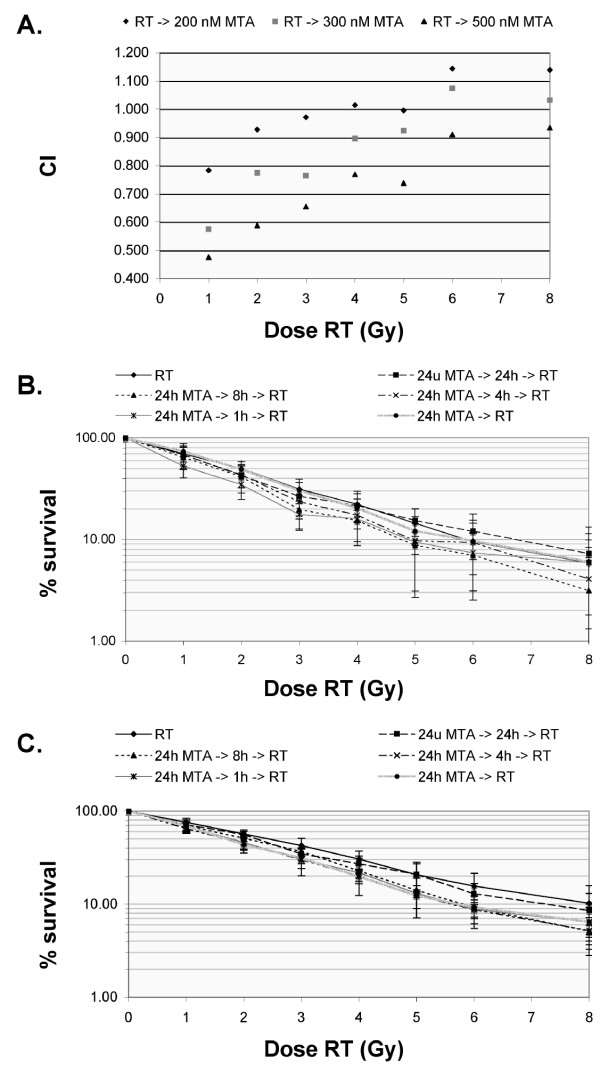
**MTA combined with irradiation**. *(A) *Combination index (CI) plots of radiation (RT) immediately followed by 24 h incubation with various concentrations of MTA in A549 cells. The plots represent the mean of at least three independent experiments. *(B-C) *Radiation dose-response curves of CAL-27 *(B) *and A549 *(C) *cells treated with 24 h MTA followed by different time intervals (24, 8, 4, 1, 0 h) and subsequent irradiation. Survival values from combination experiments were normalised to MTA cytotoxicity (see table 1 for the cytotoxic effect of MTA alone). Data points represent mean values from at least three independent experiments; *bars*, SD.

Irradiation preceding 24 h incubation with MTA resulted in an additive effect in CAL-27 cells, with DEFs ranging from 0.87 ± 0.06 to 1.00 ± 0.24 and mean CI values of 1.005 ± 0.080 to 1.039 ± 0.098. In A549 cells, DEFs indicated a slightly radioprotective effect for this treatment schedule (0.7<DEF<0.9). A closer look at the CI analysis (figure 1A) showed that the interaction was synergistic to moderately synergistic in the lower dose range and shifted towards antagonism with increasing doses of irradiation. This is also reflected in the dose-survival curves.

To examine the role of a time interval between pemetrexed and radiation treatment, CAL-27 and A549 cells were incubated with only slightly cytotoxic concentrations of MTA (i.e. 50 nM and 100 nM for resp. CAL-27 and A549) (figure 1B, C). Statistical analysis by two-way ANOVA showed that the DEF was significantly influenced by the time interval between MTA treatment and irradiation in both CAL-27 (p = 0.005) and A549 (p = 0.014) cells. A 1 h time interval between 24 h MTA and irradiation resulted in a clear radiosensitising effect in both cell lines, with DEF 1.52 ± 0.39 for A549 cells and 1.46 ± 0.21 for CAL-27 cells (which was significantly higher than the DEF for 24 h MTA immediately followed by RT, p = 0.008). In the CAL-27 cell line, radiosensitisation was also observed after a 24 h or 8 h time interval (DEF resp. 1.59 ± 0.53 and 1.23 ± 0.31), with CI analysis indicating moderate synergism at the 8 h time interval. In A549 cells, CI values increased from 0.677 ± 0.076 for the 1 h interval (synergism) to 0.832 ± 0.067 for the 24 h interval (moderate synergism). Concomitantly, DEF values gradually decreased with an increasing time interval between incubation with MTA and irradiation.

### Cell cycle distribution

**Figure 2:**
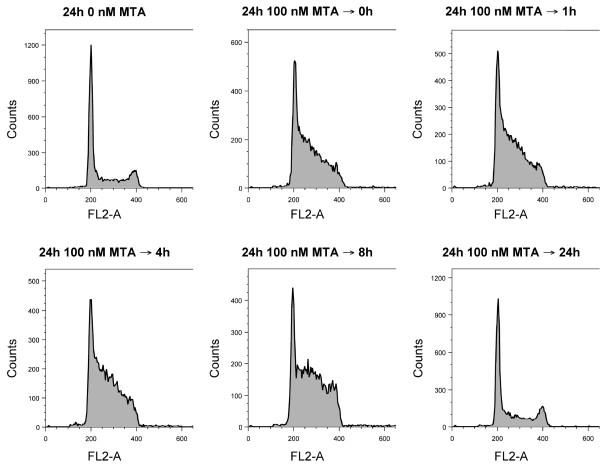
**Influence of MTA on cell cycle distribution**. DNA histograms of A549 cells at different time points after 24 h incubation with 100 nM MTA.

Table 2 summarises the cell cycle distribution of CAL-27 and A549 cells after incubation with pemetrexed for 24 h, followed by a drug free period of 0, 1, 4, 8 or 24 h. Treatment with pemetrexed for 24 h induced a significant increase in the percentage of S phase cells (control: 37.1 ± 1.3%; 24+0: 66.5 ± 6.2% in A549 cells), accompanied by a significant decrease in the number of G_0/1 _phase cells (control: 49.5 ± 3.1%; 24+0: 23.3 ± 6.1% in A549 cells). These changes in cell cycle distribution were observed for up to 8 h after drug removal, whereas drug wash out for 24 h almost restored the normal distribution (figure 2). Statistical analysis using two-way ANOVA revealed that the number of S phase cells was significantly influenced depending on the cell line (CAL-27 vs. A549), duration of drug wash out and concentration of pemetrexed (control vs. treated). *Post hoc *analysis revealed a significant difference in the percentage of S phase cells between the 24+24 schedule versus the 24+1, 24+4 and 24+8 schedules.

**Table 2:** Influence of 24 h MTA on the percentage cells in G_1_, S and G_2_/M phase.

	Condition	**G**_**1 **_**phase**			S phase			**G**_**2**_**/M phase**		
		Mean	SD		mean	SD		mean	SD	
**CAL-27**	24 h 0 nM MTA	49.3	2.2		34.3	2.5		10.8	2.4	
	24 h 50 nM MTA → 0 h	30.4	13.7		56.2	14.1		8.9	3.8	
	24 h 50 nM MTA → 1 h	30.9	12.9		54.5	12.3	*	9.5	4.0	
	24 h 50 nM MTA → 4 h	30.1	9.8	*	53.5	7.5	*	10.8	1.6	
	24 h 50 nM MTA → 8 h	29.0	0.1	*	49.1	2.4	*	15.2	2.0	
	24 h 50 nM MTA → 24 h	41.5	7.9		38.9	6.8		9.2	8.1	

**A549**	24 h 0 nM MTA	49.5	3.1		37.1	1.3		11.3	0.8	
	24 h 100 nM MTA → 0 h	23.3	6.1	*	66.5	6.2	*	7.2	1.5	*
	24 h 100 nM MTA → 1 h	25.4	4.2	*	65.8	4.6	*	5.4	1.1	*
	24 h 100 nM MTA → 4 h	23.1	5.5	*	68.0	6.3	*	7.9	2.9	
	24 h 100 nM MTA → 8 h	16.4	0.4	*	71.3	1.5	*	8.5	2.1	
	24 h 100 nM MTA → 24 h	57.1	18.8		38.2	0.8		12.4	0.9	

Cell cycle analysis was performed 0, 1, 4, 8 or 24 h after drug wash out. *: p < 0.05 vs. untreated control

### Combining pemetrexed with gemcitabine

Considering the lack of concordance among previous preclinical studies on pemetrexed-gemcitabine combinations with regard to the preferable sequence of drug administration, we first investigated different combination protocols, before starting triple treatment with irradiation.

**Figure 3:**
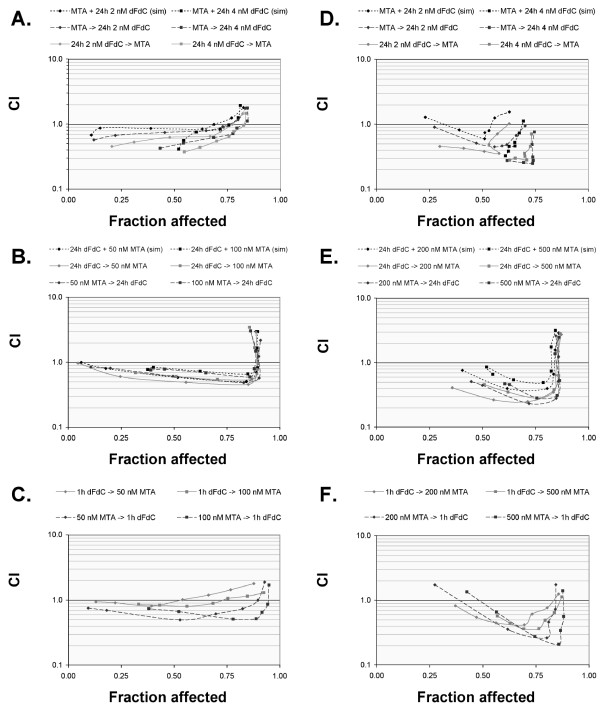
**Combination index (CI) plots of MTA - dFdC combinations in CAL-27 *(A-C) *and A549 *(D-F) *cells**. Cells were treated with a concentration range of MTA for 24 h (0-100 nM for CAL-27 cells; 0-2000 nM for A549 cells) combined with a constant concentration of dFdC for 24 h (2 or 4 nM) *(A, D)*. Alternatively, cells were incubated with a concentration range of dFdC (0-100 nM for 24 h *(B, E) *or 0-5 μM for 1 h *(C, F)*) combined with a constant concentration of MTA (50 or 100 nM for CAL-27; 200 or 500 nM for A549). The plots represent the mean of at least three independent experiments.

The interaction between pemetrexed and gemcitabine was investigated using three different schemes (cfr. Methods). Simultaneous exposure to 24 h MTA and 24 h dFdC resulted in a significantly higher IC_50 _value in both CAL-27 and A549 cells (e.g. for A549 IC_50 _for 24 h dFdC: 3.64 ± 0.27 nM; IC_50 _for 24 h dFdC + 24 h 200 nM MTA: 7.48 ± 1.48 nM) (table 3). The sequential exposure of cell lines to 24 h pemetrexed followed by 24 h gemcitabine caused an increase in the IC_50 _values too, which was significant for CAL-27 cells (e.g. IC_50 _for 24 h MTA: 119 ± 16 nM; IC_50 _for 24 h MTA → 24 h 2 nM dFdC: 146 ± 12 nM). In contrast, the inverted sequence induced a decrease in the IC_50 _value of MTA in CAL-27 cells (IC_50 _for 24 h wash out → 24 h MTA: 198 ± 15 nM; IC_50 _for 24 h 2 nM dFdC → 24 h MTA: 132 ± 15 nM). However, in A549 cells, 24 h gemcitabine followed by 24 h pemetrexed induced an increase in IC_50 _values in comparison with monotherapy with gemcitabine or pemetrexed alone. Calculation of the combination index (figure 3A, B, D, E) showed antagonistic interactions at the higher cytotoxic range. At fraction effects between 0.25 and 0.75, all schedules (simultaneous and sequential) of 24 h gemcitabine and 24 h MTA demonstrated synergistic to additive effects in both CAL-27 and A549; however, though the differences were not marked, the sequence 24 h dFdC → 24 h MTA seemed to be the most effective treatment schedule.

**Table 3:** IC_50 _and combination index (CI) values for MTA - dFdC combinations in CAL-27 and A549 cells.

Condition	CAL-27							A549						
	**IC**_**50 **_**(nM)**	SD		CI	SD				**IC**_**50 **_**(nM)**	SD		CI	SD	
24 h MTA → 24 h wash out	118.53	16.37							569.47	120.60				
24 h wash out → 24 h MTA	198.25	14.51							1576.86	349.19				
														
24 h MTA + 24 h 2 nM dFdC → 24 h wash out	147.44	18.68		1.038	0.378				1689.43	1289.82		1.003	0.352	
24 h MTA + 24 h 4 nM dFdC → 24 h wash out	240.31	49.10	†	0.957	0.510				9120.46	1499.06	†	0.569	0.271	**
24 h MTA → 24 h 2 nM dFdC	146.31	12.08	†	0.943	0.403				452.53	177.35		0.632	0.213	**
24 h MTA → 24 h 4 nM dFdC	130.60	29.92		0.858	0.461	*			1069.95	1035.22		0.373	0.190	**
24 h 2 nM dFdC → 24 h MTA	132.00	14.59	†	0.779	0.341	*			1322.54	833.14		0.546	0.237	**
24 h 4 nM dFdC → 24 h MTA	172.68	42.09		0.739	0.373	*			6076.56	3648.50		0.384	0.154	**
														
24 h dFdC → 24 h wash out	4.35	0.39							3.64	0.27				
24 h wash out → 24 h dFdC	5.87	0.17							5.30	0.20				
														
24 h dFdC + 24 h 50 nM MTA → 24 h wash out	4.25	0.37		1.141	0.858									
24 h dFdC + 24 h 100 nM MTA → 24 h wash out	5.82	0.68	†	1.201	0.843									
24 h dFdC + 24 h 200 nM MTA → 24 h wash out									7.48	1.04	†	1.112	0.972	
24 h dFdC + 24 h 500 nM MTA → 24 h wash out									19.40	11.06		1.167	0.967	
24 h dFdC → 24 h 50 nM MTA	3.83	0.07		1.073	0.872									
24 h dFdC → 24 h 100 nM MTA	3.61	0.20		1.179	1.071									
24 h dFdC → 24 h 200 nM MTA									4.90	1.79		0.873	0.924	*
24 h dFdC → 24 h 500 nM MTA									7.08	1.87		0.921	0.942	
24 h 50 nM MTA → 24 h dFdC	4.88	0.29	†	0.961	0.591									
24 h 100 nM MTA → 24 h dFdC	5.89	0.83		1.158	0.870									
24 h 200 nM MTA → 24 h dFdC									6.96	2.52		0.830	0.848	*
24 h 500 nM MTA → 24 h dFdC									14.81	10.54		0.838	0.806	*
														
1 h dFdC → 24 h wash out	478.76	149.90							533.46	197.64				
24 h wash out → 1 h dFdC	495.55	130.97							569.36	96.66				
														
1 h dFdC → 24 h 50 nM MTA	571.61	262.49		1.154	0.349									
1 h dFdC → 24 h 100 nM MTA	386.35	92.33		0.980	0.185									
1 h dFdC → 24 h 200 nM MTA									421.84	237.54		0.693	0.287	**
1 h dFdC → 24 h 500 nM MTA									407.32	187.49		0.570	0.265	**
24 h 50 nM MTA → 1 h dFdC	271.36	38.36		0.880	0.466	*								
24 h 100 nM MTA → 1 h dFdC	166.12	16.64	†	0.803	0.413	*								
24 h 200 nM MTA → 1 h dFdC									215.44	21.72	†	0.880	0.682	*
24 h 500 nM MTA → 1 h dFdC									217.14	24.80	†	0.681	0.494	**

†: p < 0.05 vs. IC_50 _of the corresponding drug alone; *: moderate synergism (0.7<CI<0.9); **: synergism (CI<0.7).

**Figure 4:**
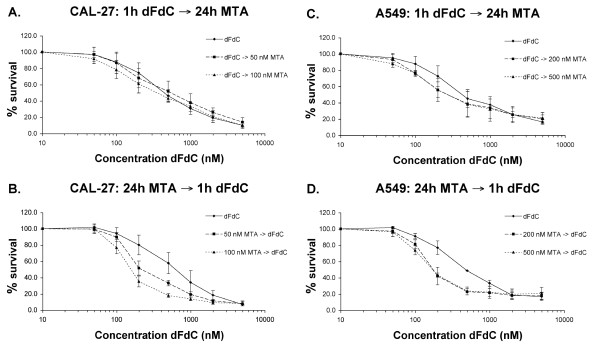
**Inhibitory effect of 24 h MTA - 1 h dFdC combinations on cell survival of CAL-27 *(A, B) *and A549 *(C, D) *tumour cells**. Survival curves were corrected for the cytotoxic effect of 24 h MTA alone. In CAL-27, 24 h incubation with 50 or 100 nM MTA resulted in resp. 90 ± 7% and 73 ± 8% survival. In A549, 24 h incubation with 200 or 500 nM MTA resulted in resp. 73 ± 10% and 50 ± 7% survival. Data points represent mean values from at least three independent experiments; *bars*, SD.

The dose-effect curves of sequential combination of 1 h dFdC and 24 h MTA (figure 4) show that pemetrexed enhanced the growth inhibition of 1 h gemcitabine in both cell lines. Calculation of the CI value at fraction effects between 0.25 and 0.75 (figure 3C, F) revealed synergism in A549 cells, yet additivity in the CAL-27 cell line. In contrast, the reverse sequence (i.e. 24 h MTA → 1 h dFdC) induced a synergistic interaction in both cell lines, with a significant decrease in IC_50 _values (table 3). For example, sequential exposure to 200 nM MTA followed by 1 h gemcitabine considerably reduced the IC_50 _of 1 h gemcitabine from 569 ± 97 nM to 215 ± 22 nM. Taking into account IC_50 _values calculated from the survival curves as well as the CI analysis for both CAL-27 and A549 cells, the degree of synergism obtained with the 24 h MTA → 1 h dFdC sequence was remarkably greater than that observed with the other schedules investigated.

### Combining pemetrexed with gemcitabine and irradiation

CAL-27 and A549 cells were irradiated in combination with different schedules of pemetrexed and gemcitabine pretreatment. For all schedules included, treatment of the tumour cells with gemcitabine, pemetrexed and radiation produced growth inhibition that was additive to synergistic in comparison to monotherapy with gemcitabine, pemetrexed or irradiation alone (table 4). For example, simultaneous administration of MTA and dFdC for 24 h prior to irradiation resulted in CAL-27 and A549 cells in a dose enhancement factor of resp. 1.53 ± 0.27 and 1.49 ± 0.28. Both sequential schedules with 1 h dFdC preceding or following 24 h MTA showed radiosensitising potential (DEF 1.29-1.56), with CI values indicating moderate synergism in CAL-27 cells and synergism in A549 cells. Especially 24 h MTA followed by 1 h dFdC and irradiation seemed interesting to us, since this scheme resulted in DEFs around 1.50 in both cell lines. Moreover, the percentage of growth inhibition induced by this drug combination (survival rate 65 ± 11% for CAL-27; 49 ± 8% for A549) was significantly lower than the cytotoxic effect of MTA alone and that of dFdC alone. This can be explained by the synergistic interaction observed with the 24 h MTA → 1 h dFdC combination (without radiotherapy).

**Table 4:** DEF, CI, ID_50_, SF_2 _and % survival (representing the cytotoxic effect of treatment with MTA and/or dFdC alone, 0 Gy) for MTA - dFdC - irradiation (RT) combinations in CAL-27 and A549 cells.

	Condition	DEF			CI			**ID**_**50**_			**SF**_**2**_			% survival		
		mean	SD		mean	SD		mean	SD		mean	SD		mean	SD	
**CAL-27**	RT							1.94	0.47		48.37	9.31		100%	0%	
	24 h 50 nM MTA → RT	1.04	0.23		1.095	0.127		1.97	0.45		49.25	9.71		97%	11%	
	24 h 50 nM MTA → 24 h → RT	1.59	0.53		0.916	0.063		1.71	0.66		43.37	12.35		70%	21%	
	24 h 50 nM MTA → 1 h → RT	1.46	0.21		0.933	0.073		1.21	0.35		32.45	8.43		98%	4%	
																
	24 h 2 nM dFdC → RT	1.47	0.09		0.888	0.180	*	1.72	0.10		49.25	9.71		96%	1%	
	24 h 2 nM dFdC → 24 h → RT	1.04	0.16		1.090	0.101		2.43	0.08		58.31	2.96		84%	18%	
	1 h 100 nM dFdC → 24 h → RT	1.56	0.46		0.653	0.108	**	1.32	0.25		34.09	5.34		91%	24%	
	1 h 50 nM dFdC → RT	1.07	0.19		0.917	0.124		1.69	0.64		44.73	14.77		98%	14%	
																
	24 h 2 nM dFdC + 24 h 50 nM MTA → RT	1.53	0.27	†	0.960	0.267		1.66	0.09		42.27	1.03		98%	11%	
	24 h 2 nM dFdC → 24 h 50 nM MTA → RT	1.38	0.09		0.949	0.106		1.85	0.34		46.93	6.81		75%	4%	†
	24 h 50 nM MTA → 24 h 2 nM dFdC → RT	1.78	0.14		0.705	0.097	*	1.42	0.06		36.83	3.36		64%	22%	
																
	1 h 100 nM dFdC → 24 h 50 nM MTA → RT	1.56	0.37	†	0.801	0.094	*	1.29	0.20	†	34.65	4.07	†	71%	15%	†
	24 h 50 nM MTA → 1 h 50 nM dFdC → RT	1.43	0.57		0.858	0.175	*	1.30	0.45		34.93	10.25		65%	11%	†;††

**A549**	RT							2.41	0.39		56.26	6.18		100%	0%	
	24 h 100 nM MTA → RT	1.29	0.25		0.674	0.053	**	1.77	0.36		45.39	7.15		87%	11%	
	24 h 100 nM MTA → 24 h → RT	0.98	0.15		0.838	0.070	*	2.16	0.11		52.70	2.00		84%	16%	
	24 h 100 nM MTA → 1 h → RT	1.52	0.39		0.681	0.084	**	1.85	0.51		46.28	9.78		85%	11%	
	24 h 2 nM dFdC → RT	1.55	0.24		0.538	0.057	**	1.25	0.08		34.55	2.01		71%	16%	
	24 h 2 nM dFdC → 24 h → RT	0.99	0.31		0.703	0.118	*	2.04	0.40		50.19	6.96		65%	4%	
	1 h 100 nM dFdC → 24 h → RT	1.23	0.23		0.606	0.073	**	1.97	0.24		49.28	4.36		67%	13%	
	1 h 50 nM dFdC → RT	1.49	0.39		0.831	0.068	*	1.92	0.49		47.99	8.72		96%	5%	
																
	24 h 2 nM dFdC + 24 h 100 nM MTA → RT	1.49	0.28		0.564	0.075	**	1.32	0.30		36.11	6.47		66%	23%	
	24 h 2 nM dFdC → 24 h 100 nM MTA → RT	0.94	0.27		0.585	0.101	**	2.21	0.80		52.39	11.98		47%	14%	
	24 h 100 nM MTA → 24 h 2 nM dFdC → RT	1.17	0.24		0.500	0.073	**	1.69	0.36		44.34	7.16		48%	16%	†
																
	1 h 100 nM dFdC → 24 h 100 nM MTA → RT	1.29	0.27		0.513	0.053	**	1.88	0.25		47.68	4.45		48%	12%	†
	24 h 100 nM MTA → 1 h 50 nM dFdC → RT	1.52	0.58		0.534	0.083	**	1.92	0.50		47.93	9.71		49%	8%	†;††

†: p < 0.05 vs. corresponding schedule of MTA and RT; ††: p < 0.05 vs. corresponding schedule of dFdC and RT; *: moderate synergism (0.7<CI<0.9); **: synergism (CI<0.7).

## Discussion

Today, the mainstay of cancer treatments consists of surgery, radiotherapy and/or chemotherapy. In daily practice, the combination of radiotherapy and chemotherapy has become a standard treatment and it is associated with improved survival rates in many tumours, thereby favouring multimodal strategies in tumour therapy. A multitude of potential interaction mechanisms between radiotherapy and chemotherapy, including radiosensitisation of tumour cells through drug exposure, may improve treatment results [38]. Given the reported radiosensitising potential of both gemcitabine [39] and pemetrexed [23], this paper, for the first time, describes a preclinical study evaluating the triple combination of pemetrexed, gemcitabine and irradiation.

Concentration-dependent growth inhibition by single agent treatment with gemcitabine or pemetrexed was observed in both A549 lung carcinoma and CAL-27 head and neck carcinoma cell lines, with IC_50 _values <1.0 μM in all cases, which is well below the mean peak plasma concentration of both drugs achievable in patients [40,41].

The interaction between pemetrexed and irradiation was examined as a potential strategy to enhance the therapeutic ratio of combined-modality cancer treatment. However, incubation of CAL-27 or A549 cells with 24 h pemetrexed immediately preceding or following irradiation (0-8 Gy) was unable to produce any significant radiosensitisation of the tumour cells. In contrast, Bischof et al demonstrated that a concomitant exposure to ionising radiation and moderately toxic concentrations of pemetrexed (106 nM, 70% survival) inhibited clonogenic survival in excess of independent toxicities in all four human tumour carcinoma cell lines tested, with enhancement ratios ranging from 1.2 (HeLa cervix carcinoma and MCF-7 breast carcinoma cells) to 1.6 (LXI lung carcinoma cells). In WiDr colon carcinoma cells, significant radiosensitisation (DEF 1.8) was only noticed at higher pemetrexed concentrations (636 nM, 85% survival), with a DEF of 1.1 when cells were pretreated with 106 nM pemetrexed (no cytotoxic effect) [23].

As the timing of irradiation relative to drug application may play an important role in combined modality treatments, tumour cells were irradiated at different time intervals between 24 h pemetrexed treatment and irradiation. Overall, including a time interval between pemetrexed exposure and irradiation seemed favourable to pemetrexed immediately preceding or following radiotherapy, with DEFs up to 1.6 for CAL-27 cells. No readily observable tendency in cell killing was shown over the different time intervals. For both CAL-27 and A549 cells, a 1 h time interval resulted in a clear radiosensitising effect (DEF 1.5). Similarly, no substantial variation in survival fraction could be observed in WiDr colon carcinoma cells when the interval between the start of 2 h pemetrexed exposure and irradiation was varied from -4 h to +10 h [23]. This finding led to the hypothesis that pemetrexed possibly exerts its radiosensitising potential very rapidly and that this effect pertains after drug removal for an extended period of time (at least 8 hours). Interestingly, a different behavior has been reported for gemcitabine, where the radiosensitising potential gradually decreased with an increasing time interval [34].

Our findings in CAL-27 and A549 cells, showing S phase accumulation when cells were treated with only slightly toxic concentrations of pemetrexed for 24 h, are consistent with previous data in the A549 cell line [22,42]. The S phase accumulation was observed for up to 8 h after drug removal, yet disappeared after 24 h wash out. This implies that the differences in radiosensitisation could not be explained by the pemetrexed-induced S phase accumulation (see also table 1 and 2). Correspondingly, the study by Bischof et al also excluded the S phase enrichment as the primary mechanism for radiosensitisation by pemetrexed [23]. Moreover, tumour cell apoptosis was not found to be responsible for pemetrexed-induced radiosensitisation in human colon carcinoma cells [43]. Thus, the differential radiosensitisation induced by pemetrexed cannot be explained at present. A number of causes appear conceivable (such as differences in drug toxicity levels, growth characteristics of the cell lines investigated, levels of drug-inhibited enzymes, or intracellular pemetrexed polyglutamation), and further assessment of the molecular mechanisms underlying the radiosensitising potential of pemetrexed seems crucial.

Our study aimed at investigating the triple combination of gemcitabine, pemetrexed and irradiation. However, a recommended protocol for gemcitabine/pemetrexed combinations differed among previously published *in vitro *studies and there was generally no agreement with regard to the preferable treatment schedule. The drug combination has been examined *in vitro *with different human tumour cell lines (including colon, bladder and pancreatic cancer, NSCLC, and malignant pleural mesothelioma), resulting in controversial schedule-dependent interactions. Though simultaneous drug administration is the more frequently used and most practical clinical regimen, results from the present and previous *in vitro *studies showed that simultaneous exposure to these two antimetabolites did not significantly increase cell kill and thus probably will not improve the clinical therapeutic effect [42,44,45]. Conversely, we observed that sequential exposure produced a greater cytotoxic effect than that exerted by single-agent use or simultaneous exposure. In particular, as shown by the IC_50 _values calculated from survival curves as well as the results from CI analysis in both A549 and CAL-27 cells, a higher synergistic interaction was obtained by pretreatment with 24 h pemetrexed followed by 1 h gemcitabine (24 h MTA → 1 h dFdC) in comparison with the other schedules investigated. These findings are in agreement with previous reported observations in the A549 NSCLC cell line by Giovannetti et al [22,30]; for the CAL-27 SCCHN cell line, no previous data are available. In the clinic, a phase I trial in patients with advanced solid tumours suggested that the sequence of gemcitabine administered on days 1 and 8 with pemetrexed administered on day 8, 90 minutes after gemcitabine was well tolerated and recommended for further study [46]. However, a few years later, the same research group conducted a phase II trial of three schedules of pemetrexed and gemcitabine as front-line therapy for advanced NSCLC. In this trial, the pemetrexed-gemcitabine schedule was less toxic compared with other sequences and, by obtaining a confirmed response rate of 31%, was the only schedule that met the protocol-defined efficacy criteria [47]. As such, both preclinical and clinical data support the sequential pemetrexed-gemcitabine schedule in NSCLC.

Concerning the molecular basis for pemetrexed-gemcitabine interactions, it has been suggested that the favourable modulation of the cell cycle by pemetrexed may be considered as one of the most important mechanisms underlying the synergistic interaction in the 24 h MTA → 1 h dFdC sequence [22]. Because gemcitabine is an S phase specific drug, the increase in its activity in this schedule may be the result of the S phase accumulation induced by pemetrexed, which potentially facilitates incorporation of 2',2'-difluoro-deoxycytidine triphosphate into the DNA. As the cell cycle modulation by pemetrexed lasted for several hours after drug removal, but disappeared after 24 h, this may explain why the 24 h MTA → 1 h dFdC seems preferable to the 24 h MTA → 24 h dFdC schedule.

In A549 cells, it has been demonstrated that pemetrexed, at its IC_50 _and IC_75 _levels, significantly upregulated the hENT1 carrier, potentially facilitating gemcitabine cytotoxicity [22]. Moreover, being an inhibitor of de novo purine biosynthesis (because of the blockade of the key enzyme GARFT), pemetrexed was shown to increase the expression of dCK as a compensatory mechanism [22]. The dCK activity of untreated A549 and CAL-27 cells was reported to be highly comparable (resp. 6.02 and 5.02 nmol/h/mg protein) and a weak positive correlation between dCK activity and the radiosensitising effect of gemcitabine has been reported [48], suggesting that enhancement of hENT1 and dCK expression by pemetrexed in the pemetrexed → gemcitabine sequence strongly supports this combination.

In addition, several studies showed that TS expression is significantly correlated with pemetrexed sensitivity both in a preclinical and clinical setting [22,49]. Functional inactivity and mutations of p53 were shown to differentially affect the expression and activity of TS [50], potentially influencing the response of A549 (wt p53) and CAL-27 (mt p53) cells to pemetrexed-based treatment. Nevertheless, different conclusions regarding the relationship between functional p53 status and sensitivity to pemetrexed have been obtained, possibly depending on the phenotypic/genotypic background of the model system used [29,51-53]. Similarly, the role of p53 on the ability of gemcitabine to induce a cytotoxic and radiosensitising effect is not yet completely elucidated [6,54,55], making further mechanistic unravelling of the pemetrexed-gemcitabine-radiation combination highly warranted.

When combining pemetrexed and gemcitabine with irradiation, the 24 h MTA → 1 h dFdC → RT regimen showed radiosensitising potential in both cell lines (DEF 1.4 for CAL-27; 1.5 for A549). Other pemetrexed/gemcitabine schedules in combination with radiation also produced additive to synergistic growth inhibition in comparison to monotherapy, and the corresponding DEFs were not significantly different from these obtained with 24 h MTA → 1 h dFdC → RT. However, given the synergistic interaction between 24 h pemetrexed and 1 h gemcitabine, the 24 h MTA → 1 h dFdC → RT turned out to be the preferred schedule for combined administration with radiotherapy in our preclinical model system.

## Conclusions

This study characterises, for the first time, the interactions between gemcitabine, pemetrexed and radiotherapy. Preliminary results from our *in vitro *model suggest that the sequence 24 h MTA → 1 h dFdC → RT is the most rational design. Further in depth mechanistic unravelling of the pemetrexed-gemcitabine-radiation combination is certainly needed. As extrapolation of *in vitro *data to the clinic should be considered with caution, the experiments provide a strong experimental basis for future development of this triple combination in an *in vivo *setting.

## Competing interests

The authors declare that they have no competing interests. Pemetrexed and gemcitabine were kindly provided by Eli Lilly (Indianapolis, USA).

## Authors' contributions

AW participated in the design of the study, performed the experiments and the statistical analysis and drafted the manuscript. GP and HL participated in the cell survival experiments and performed cell culture. MB participated in the acquisition of data. PM was involved in the irradiation experiments. BP, FL and JBV participated in the conception, design, and coordination of the study, and revised the manuscript critically. All authors read and approved the final manuscript.

## Pre-publication history

The pre-publication history for this paper can be accessed here:

http://www.biomedcentral.com/1471-2407/10/441/prepub
